# Dyspepsia Caused by Tight Lacing

**Published:** 1881-07

**Authors:** 


Dyspepsia Caused by Tight Lacing.—Dyce Durkworth, M.D., in *The Practitioner*, says: I find many cases of dyspepsia in women yield quickly to the use of proper stays. Again and again I have known chronic vomiting in young girls to be due solely to tight stays. Palpitation and dyspnœa, not due to anæmia, are frequently caused by bad stays. The worst cases naturally occur in young women who are inclined to *embonpoint*, and whether this be constitutional or aggravated, as is that condition by anæmia, the obese tendency commonly both adds to the compression and gives cause to the wearer to increase her troubles in the efforts to retain (what she conceives to be) shapely proportions.

